# HiCNN2: Enhancing the Resolution of Hi-C Data Using an Ensemble of Convolutional Neural Networks

**DOI:** 10.3390/genes10110862

**Published:** 2019-10-30

**Authors:** Tong Liu, Zheng Wang

**Affiliations:** Department of Computer Science, University of Miami, 1365 Memorial Drive, P.O. Box 248154, Coral Gables, FL 33124, USA; tong.liu@miami.edu

**Keywords:** Hi-C, 3D genome, super-resolution, convolutional networks, resolution enhancement

## Abstract

We present a deep-learning package named HiCNN2 to learn the mapping between low-resolution and high-resolution Hi-C (a technique for capturing genome-wide chromatin interactions) data, which can enhance the resolution of Hi-C interaction matrices. The HiCNN2 package includes three methods each with a different deep learning architecture: HiCNN2-1 is based on one single convolutional neural network (ConvNet); HiCNN2-2 consists of an ensemble of two different ConvNets; and HiCNN2-3 is an ensemble of three different ConvNets. Our evaluation results indicate that HiCNN2-enhanced high-resolution Hi-C data achieve smaller mean squared error and higher Pearson’s correlation coefficients with experimental high-resolution Hi-C data compared with existing methods HiCPlus and HiCNN. Moreover, all of the three HiCNN2 methods can recover more significant interactions detected by Fit-Hi-C compared to HiCPlus and HiCNN. Based on our evaluation results, we would recommend using HiCNN2-1 and HiCNN2-3 if recovering more significant interactions from Hi-C data is of interest, and HiCNN2-2 and HiCNN if the goal is to achieve higher reproducibility scores between the enhanced Hi-C matrix and the real high-resolution Hi-C matrix.

## 1. Introduction

The population-cell Hi-C technique [1] can capture genome-wide intra- and inter-chromosomal contacts, which provide proximity information of the DNA and can be used to reconstruct the three-dimensional (3D) structures of chromosomes [2,3,4], define topologically associated domains (TADs) [5,6,7], and reveal significant genomic interactions [8,9]. In the past decade, researchers have conducted Hi-C experiments for different species at different resolutions [1,6,9,10]. It has been shown that high-resolution Hi-C data are essential for studies of the 3D genome [9,10]. However, to experimentally obtain high-resolution (e.g., 5 kb) Hi-C data, researchers need to generate more than one billion paired-end reads [9], which may incur a high sequencing cost. Moreover, the whole process of the in situ Hi-C protocol [9] is time-consuming. Therefore, computational methods for resolution enhancement of Hi-C data are indispensable. Recently, a single-cell Hi-C technique was developed that can be used to reveal cell-to-cell variability [11,12]. Bioinformatics tools have been developed to remove systematic biases existing in the single-cell Hi-C data [13] and reconstruct 3D chromosomal structures based on single-cell Hi-C data [14]. However, in this research, we only focus on enhancing population-cell Hi-C data.

Given a sparse n×n Hi-C contact matrix, a resolution enhancement method can increase the intensity of the sparse (or so-called “low-resolution” in this study) matrix and output an enhanced n×n Hi-C contact matrix, in which the 2D contact patterns and Hi-C peaks are much more clearly presented than in the input low-resolution matrix. Moreover, this computational tool can also be used to increase the ideal resolution that makes the Hi-C data useful for indicating contact patterns and significant interactions. For example, given an n×n  Hi-C contact matrix, we can double its resolution to make it a 2n×2n matrix. However, this may make the matrix very sparse and cause the contact patterns and significant contacts to be blurred or disappear. The resolution enhancement tool can take a sparse 2n×2n matrix as input and then output a matrix that is also 2n×2n in size but with enhanced intensity, in which the contact patterns and significant interactions (i.e., Hi-C peaks) are clearly depicted. Although not the same, there are other bioinformatics problems facing a similar challenge. For example, in [15] the authors used convolutional neural networks to predict DNA sequences for the missing/uncertain parts of corrupted DNA sequences of extinct organisms.

HiCPlus [16] first used a three-layer convolutional neural network (ConvNet) to enhance the resolution of Hi-C data from low-resolution Hi-C data. It has been shown that HiCPlus can achieve better performance than two traditional regression methods (i.e., Gaussian smoothing and random forest) [16]. HiCPlus-enhanced high-resolution Hi-C data are even more similar to experimental high-resolution data than those high-resolution data gathered from replicate experiments. However, over the past few years multiple techniques and convolutional neural networks have been developed in the field of image super-resolution that have achieved better performance, such as local residual learning [17], global residual learning [18], a mixture of deep networks [19], and residual dense networks [20]. Therefore, there is room to improve the three-layer ConvNets that HiCPlus uses.

HiCNN [21] has achieved better performance compared with HiCPlus by using a deeper convolutional network (54 layers) which implements global and local residual learning [18,22]. In order to further improve the performance of HiCNN, we have tried to increase the number of layers to 104 by implementing 50 local residual learning blocks. However, the performance was still similar to that of the 54-layer HiCNN [19], which showed the limited improvement room for the learning architecture used in HiCNN. Therefore, in this research, we designed and benchmarked different types of deep learning architectures and different ways of combining these architectures.

Recently, generative adversarial networks (GANs) have been used for single-image super-resolution [23], which can recover photo-realistic texture from down-sampled images. Unlike individual convolutional networks, GANs need two independent architectures: the generator and discriminator. The generator is responsible for generating images that can fool the discriminator, whereas the discriminator is trained to distinguish faked images from the generator. The GAN techniques can also be used in 3D genome analysis. For example, the bioinformatics tool hicGAN [24] used GAN to enhance the resolution of Hi-C data. 

In this study, we present HiCNN2 for improving the resolution of Hi-C data. HiCNN2 is a package of three methods, each with a different deep learning architecture: HiCNN2-1 contains one type of ConvNet; HiCNN2-2 consists of an ensemble of two different types of ConvNets; and HiCNN2-3 uses an ensemble of three different types of ConvNets. In total, there are three different types of ConvNets designed in this study: the first type of ConvNet not only implements global and local residual learning but also concatenates hierarchical features from each of the local residual learning blocks; the second type of ConvNet is a modified version of VDSR (a very deep convolutional network for accurate image super-resolution) [18]; and the third type of ConvNet is the same as the three-layer network used in HiCPlus. Our evaluation results indicate that the three methods in HiCNN2 perform better than HiCPlus and HiCNN in terms of predicting high-resolution Hi-C contact counts and recovering significant Hi-C interactions. HiCNN2 is freely available at http://dna.cs.miami.edu/HiCNN2/.

## 2. Materials and Methods

### 2.1. Hi-C Data Acquisition and Processing

We used four Hi-C data sets in this study: the first one is from GEO GSE35156 for mouse embryonic stem (mES) cells [5], which has been used as real/experimental low-resolution (i.e., 40 kb) Hi-C data (build mm9); the second one is from GEO GSE63525 for human GM12878, IMR90, and K562 cells or cell-lines [9] (build hg19), which has been used to extract training, validation, and testing data; the third one is from GEO GSE96107 for mES cells [10], which has been used as real/experimental high-resolution (i.e., 5 kb) Hi-C data (build mm10); and the last one is for bacterial *Caulobacter crescentus*, obtained from [25]. The coordinates of Hi-C read pairs from the first Hi-C data set were converted from build mm9 to mm10 using liftOver [26].

The Hi-C data were processed the same way as in HiCPlus [16] and HiCNN [21]. Given a predefined resolution (e.g., 10 kb), we generated a symmetric Hi-C contact matrix for each chromosome from low- or high-resolution paired-end Hi-C reads. The low-resolution Hi-C reads of the second Hi-C data set were obtained by randomly sampling experimental high-resolution Hi-C read pairs with three different down-sampling ratios (i.e., 1/8, 1/16, and 1/25). We split one Hi-C contact matrix generated from low-resolution Hi-C read pairs into thousands of 40 × 40 submatrices with overlapping indices as the input of HiCNN2. The 40 × 40 submatrices generated based on the high-resolution Hi-C contacts were used as the target values.

The training data were extracted from chromosomes 1, 3, 5, 7, and 9 in human GM12878. The validation data were extracted from chromosome 2 in human GM12878. The testing data (each of the evaluation results was generated on a different testing data set) were extracted from different chromosomes in different cell lines (GM12878, K562, IMR90, mES, and bacteria). Therefore, in total we used 26% of the chromosomes in human GM12878 for the training and validation processes. The default target resolution we used for training, validation, and testing was 10 kb, unless otherwise specified. The best model we selected for testing was the model that achieved the minimum validation loss value. 

### 2.2. Architectures of HiCNN2 Methods

**Figure 1:**
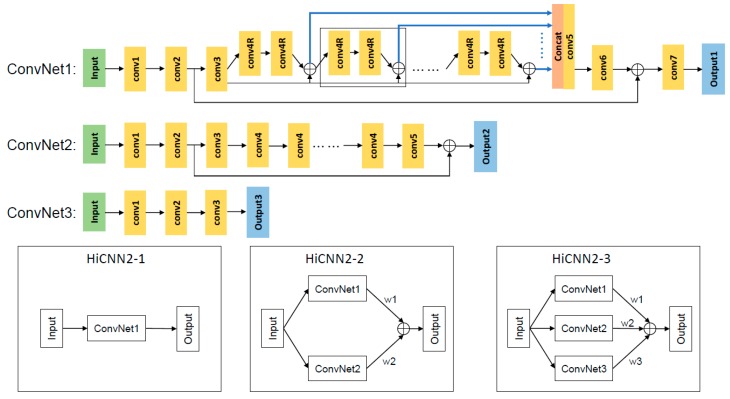
The detailed layouts of the three types of convolutional neural networks (ConvNet1, ConvNet2, and ConvNet3). ⊕ denotes element-wise addition. The dashed box in ConvNet1 highlights a local residual learning block. The blue lines in ConvNet1 denote hierarchical features. The “Concat” layer in ConvNet1 is used to concatenate all hierarchical features from each of the local residual learning blocks. HiCNN2-1 only uses ConvNet1; HiCNN2-2 is an ensemble of ConvNet1 and ConvNet2; and HiCNN2-3 is an ensemble of the three ConvNets.

We designed three architectures (HiCNN2-1, HiCNN2-2, and HiCNN2-3), shown in Figure 1, to learn the mapping between low- and high-resolution Hi-C data: (1) HiCNN2-1 only uses ConvNet1; (2) HiCNN2-2 uses an ensemble of two different convolutional networks (ConvNet1 and ConvNet2); and (3) HiCNN2-3 uses an ensemble of three distinct convolutional networks (ConvNet1, ConvNet2, and ConvNet3). 

The first ConvNet (ConvNet1 in Figure 1) has 56 layers and is an improved version of the network used in HiCNN [21], which uses both global and local learning. Compared to the network used in HiCNN, there are two main changes in ConvNet1. First, we increased the output channels of the first two layers from 8 to 64 for “conv1” and from 1 to 64 for “conv2”. Therefore, after global residual learning we use one more layer (i.e., “conv7”) to decrease the final output channel to 1. Second, we concatenated all hierarchical features from each of the local residual learning blocks (blue lines in ConvNet1 in Figure 1), which are the input of “conv5” instead of the output of the last local residual learning block. These two changes make ConvNet1/HiCNN2-1 outperform HiCNN (data shown in Section 3). The input/output channels for the seven types of layers (i.e. “conv1”, “conv2”, “conv3”, “conv4R”, “conv5”, “conv6”, and “conv7”) are 1/64, 64/64, 64/128, 128/128, 3200/1000, 1000/64, and 64/1, respectively. The input channel of “conv5” is from the concatenation of hierarchical features, which are from each of the 25 local residual learning blocks. Therefore, the input channel of “conv5” is 128 × 25 = 3200. The filter sizes for each of the seven types of layers are 13 × 13, 1 × 1, 3 × 3, 3 × 3, 1 × 1, 1 × 1, and 3 × 3, respectively. The four types of layers (“conv3”, “conv4R”, “conv5”, and “conv6”) are with zero padding of size 1.

The second ConvNet (ConvNet2 in Figure 1) has 22 layers and is a modified version of VDSR [18], which only uses global residual learning. The first two layers in ConvNet2 are the same as the first two layers in HiCNN [21]: (1) the first one (“conv1”) contains 13 × 13 filters followed by a rectified linear unit (ReLU) [27], and (2) the second one (“conv2”) contains 1 × 1 filters followed by a ReLU. The last parts (1 “conv3” layer, 18 “conv4” layers, and 1 “conv5” layer) in ConvNet2 are the same as those in VDSR [18]; all of the three types contain 3 × 3 filters with zero padding of size 1 followed by a ReLU. The input/output channels for the five types of layers (i.e. “conv1”, “conv2”, “conv3”, “conv4”, and “conv5”) are 1/8, 8/1, 1/64, 64/64, and 64/1, respectively.

The third ConvNet (ConvNet3 in Figure 1) has three layers and is the same as the network used in HiCPlus [16]. There are three layers in ConvNet3: (1) the first layer (“conv1”) contains 9 × 9 filters followed by a ReLU; (2) the second layer (“conv2”) contains 1 × 1 filters followed by a ReLU; and (3) the last layer (“conv3”) contains 5 × 5 filters followed by a ReLU. The input/output channels for the three layers (i.e. “conv1”, “conv2”, and “conv3”) are 1/8, 8/8, and 8/1, respectively.

Each of the three ConvNets takes a 40 × 40 submatrix as input and outputs a corresponding 28 × 28 submatrix, which is also the predicted high-resolution Hi-C submatrix. The final predicted outputs of the two ensembles (HiCNN2-2 and HiCNN2-3) are the weighted averaging of each ConvNet’s output:$\;Output=\;w_{1}\times Output1+\;w_{2}\times Output2$ and $Output=\;w_{1}\times Output1+\;w_{2}\times Output2+\;w_{3}\times Output3$, respectively, where the three weights (i.e., $w_{1}$, $w_{2}$, and $w_{3}$) are tuned/updated by the PyTorch learning algorithms as the other parameters in the networks. We wrote another script to concatenate the output submatrices to obtain the predicted high-resolution contact matrix for a chromosome. The loss function is the mean squared error between the output and corresponding experimental high-resolution submatrices.

### 2.3. Evaluation Metrics

We used four different metrics to evaluate HiCNN2 along with HiCPlus and HiCNN: (1) the mean squared error (MSE) between predicted and real high-resolution Hi-C data in terms of genomic distances; (2) Pearson’s or Spearman correlation coefficients between predicted and real high-resolution Hi-C data in terms of genomic distances; (3) the effectiveness of recovering significant interactions, which were detected by Fit-Hi-C [8]; and (4) HiC-spector [28], a metric for quantifying the reproducibility between the predicted and real high-resolution Hi-C contact matrices.

### 2.4. Implementations of the Convolutional Neural Networks

HiCNN2 was implemented in the same way as HiCNN [21] using Pytorch [29]. We used stochastic gradient descent (SGD) with a batch size of 256, a momentum of 0.9, and a weight decay of 0.0001. The learning rate was initially set to 0.1 and reduced by a factor of 0.1 when the mean squared error from the validation process stopped improving with 10-epoch tolerance. We used the adjustable gradient clipping technique with θ equal to 0.01 to increase the convergence speed. Compared with 12 h for training HiCNN and 28 h for training HiCPlus [21], training HiCNN2-3 took about 19 h (about 200 epochs for convergence) on a Nvidia V100 GPU with 16 GB memory; training HiCNN2-1 and HiCNN2-2 took 12 to 19 h. Even though training HiCNN2 is relatively slower than training HiCNN, HiCNN2 almost consistently outperforms HiCNN based on our evaluation shown in Section 3. Making predictions for one input matrix takes about several seconds on the same GPU. 

## 3. Results

### 3.1. Enhancing Down-Sampled Low-Resolution Hi-C Data in Human GM12878 and K562 Cells

**Figure 2:**
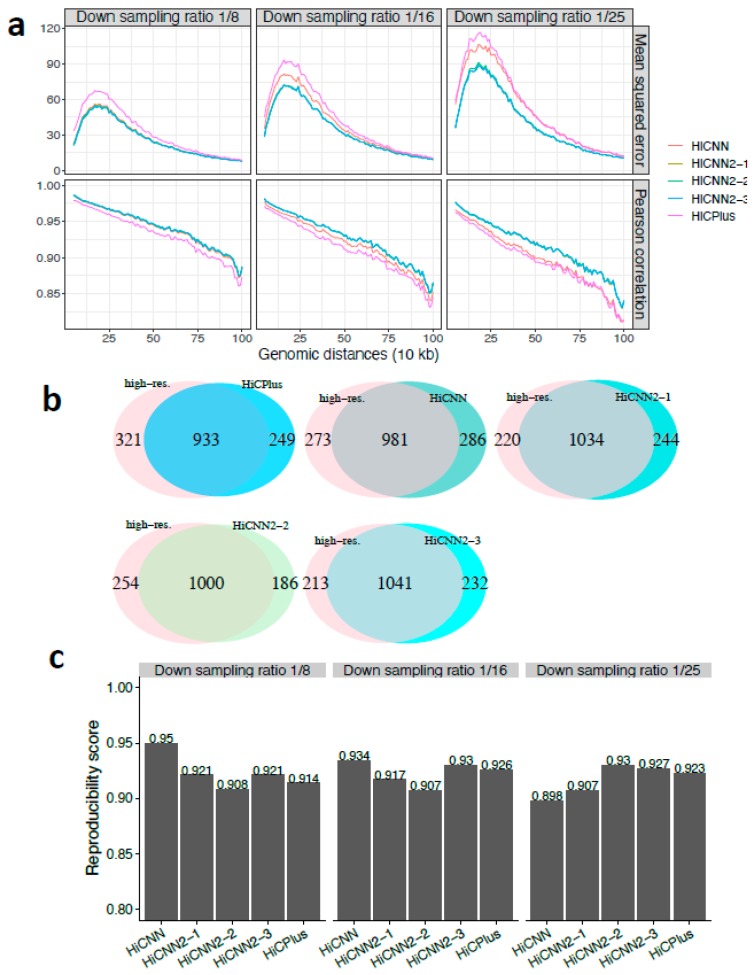
The evaluation results on chromosome 17 in human GM12878 cells between experimental high-resolution Hi-C (10 kb resolution) and each of the five predicted Hi-C data sets, namely, HiCPlus-enhanced, HiCNN-enhanced, HiCNN2-1-enhanced, HiCNN2-2-enhanced, and HiCNN2-3-enhanced: (**a**) mean squared error and Pearson’s correlations with three different down-sampling ratios (1/8, 1/16, and 1/25); (**b**) the effectiveness of recovering significant interactions (detected by Fit-Hi-C with *q*-value < 0.05) with the down-sampling ratio equal to 1/25; and (**c**) the reproducibility scores with the three down-sampling ratios.

We first evaluated the performance of HiCNN2 in comparison with HiCNN and HiCPlus in enhancing the down-sampled low-resolution Hi-C data from human cells or cell-lines. The MSE and Pearson’s correlation results on chromosome 17 in human GM12878 for HiCPlus, HiCNN, HiCNN2-1, HiCNN2-2, and HiCNN2-3 are shown in Figure 2a with three different down-sampling ratios 1/8, 1/16, and 1/25. These results indicate that (1) HiCNN2 consistently performs better than HiCNN and HiCPlus; (2) HiCNN2-1, an improved version of HiCNN, apparently achieves smaller mean squared errors and higher Pearson’s correlations than HiCNN; and (3) it is difficult to distinguish which method is better among the three HiCNN2 architectures as their performances are similar in terms of the MSE and Pearson’s correlation. 

The performances in terms of recovering significant interactions detected by Fit-Hi-C (*q*-value < 0.05, genomic distances from 50 kb to 2 Mb) are shown in Supplementary Figure S1a,b, and Figure 2b for down-sampling ratios 1/8, 1/16, and 1/25, respectively. The best two methods were HiCNN2-3 and HiCNN2-2 for ratio 1/8, HiCNN2-1 and HiCPlus for ratio 1/16, and HiCNN2-3 and HiCNN2-2 for ratio 1/25. In general, all HiCNN2 methods recovered more significant interactions than did HiCPlus and HiCNN. Moreover, we compared the significant interactions detected by Fit-Hi-C with the Hi-C peaks detected by HiCCUPS (a computational tool that searches for peaks from Hi-C data) [9]. Results with three different predefined *q*-values are shown in Table 1, indicating that compared to HiCPlus and HiCNN, the three HiCNN2 methods can recover more significant interactions that are in common with Hi-C peaks. Finally, we compared the significant interactions with the CTCF-mediated (CTCF: CCCTC-binding factor) interactions ensured by ChIA-PET [30]. There were 41, 41, 44, 39, and 44 common interactions between 4900 CTCF-mediated interacting pairs and the interactions detected from HiCPlus-enhanced, HiCNN-enhanced, HiCNN2-1-enhanced, HiCNN2-2-enhanced, and HiCNN2-3-enhanced matrices (down sampling ratio 1/25), respectively. It was found that HiCNN2-1 and HiCNN2-3 obtained more common interactions than did the other methods. 

The reproducibility scores were calculated between the experimental high-resolution Hi-C contact matrix and each of the five predicted high-resolution Hi-C matrices enhanced by HiCPlus, HiCNN, HiCNN2-1, HiCNN2-2, and HiCNN2-3. The reproducibility scores for the three different down-sampling ratios (1/8, 1/16, and 1/25) are shown in Figure 2c. Almost all of the five methods achieved high scores (>0.9). When the down-sampling ratios equaled 1/8 and 1/16, HiCNN outperformed the others and was followed by HiCNN2-3, HiCNN2-1, and HiCPlus. However, when the down-sampling ratio equaled 1/25, HiCNN2-2 achieved the highest score, followed by HiCNN2-3 and HiCPlus. In general, all of the five methods can achieve high reproducibility scores, and the improved high-resolution Hi-C data by computational methods are reliable enough to be used in practice.

**Figure 3:**
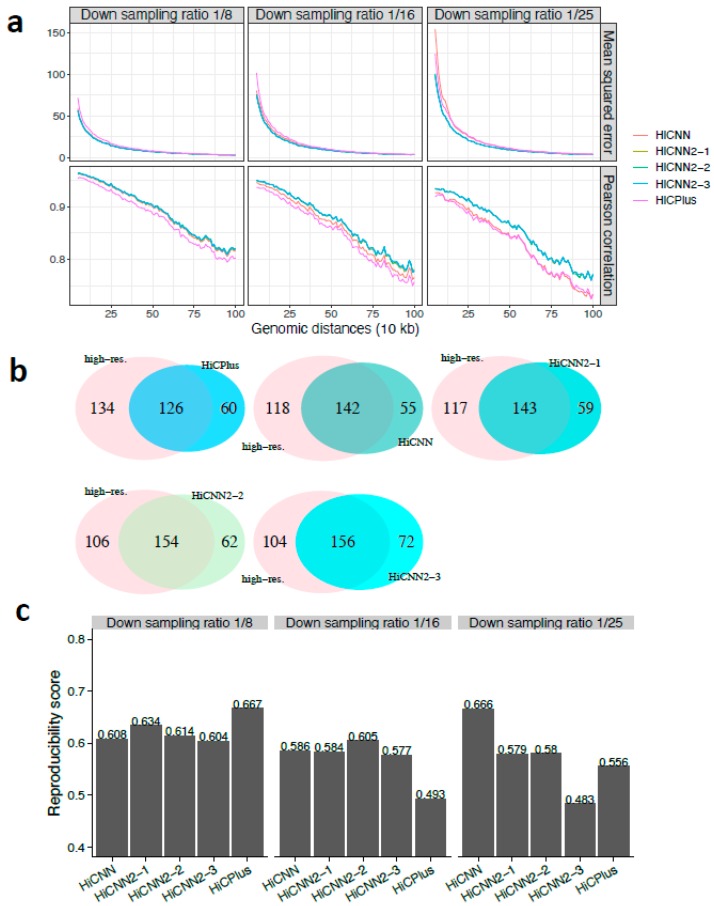
The evaluation results on chromosome 10 in human K562 cells between experimental high-resolution Hi-C (10 kb resolution) and each of the five predicted Hi-C data sets, namely, HiCPlus-enhanced, HiCNN-enhanced, HiCNN2-1-enhanced, HiCNN2-2-enhanced, and HiCNN2-3-enhanced: (**a**) mean squared error and Pearson’s correlations with three different down-sampling ratios (1/8, 1/16, and 1/25); (**b**) the effectiveness of recovering significant interactions (detected by Fit-Hi-C with *q*-value < 0.05 within the genomic distances from 50 kb to 2 Mb) with a down-sampling ratio of 1/8; and (**c**) the reproducibility scores with the three down-sampling ratios.

We next evaluated our methods on human K562 chromosome 10. The MSE and Pearson’s correlation results are shown in Figure 3a with three different down-sampling ratios, indicating that the three HiCNN2 methods consistently outperformed HiCPlus and HiCNN. The three HiCNN2 methods achieved the best effectiveness at recovering significant interactions as shown in Figure 3b and Supplementary Figure S2a,b for ratios 1/8, 1/16, and 1/25, respectively. The methods that achieved the best reproducibility scores (Figure 3c) were HiCPlus for ratio 1/8, HiCNN2-2 for ratio 1/16, and HiCNN for ratio 1/25.

### 3.2. Enhancing Down-Sampled Low-Resolution Hi-C Data in Mouse and Bacterium Cells

**Figure 4:**
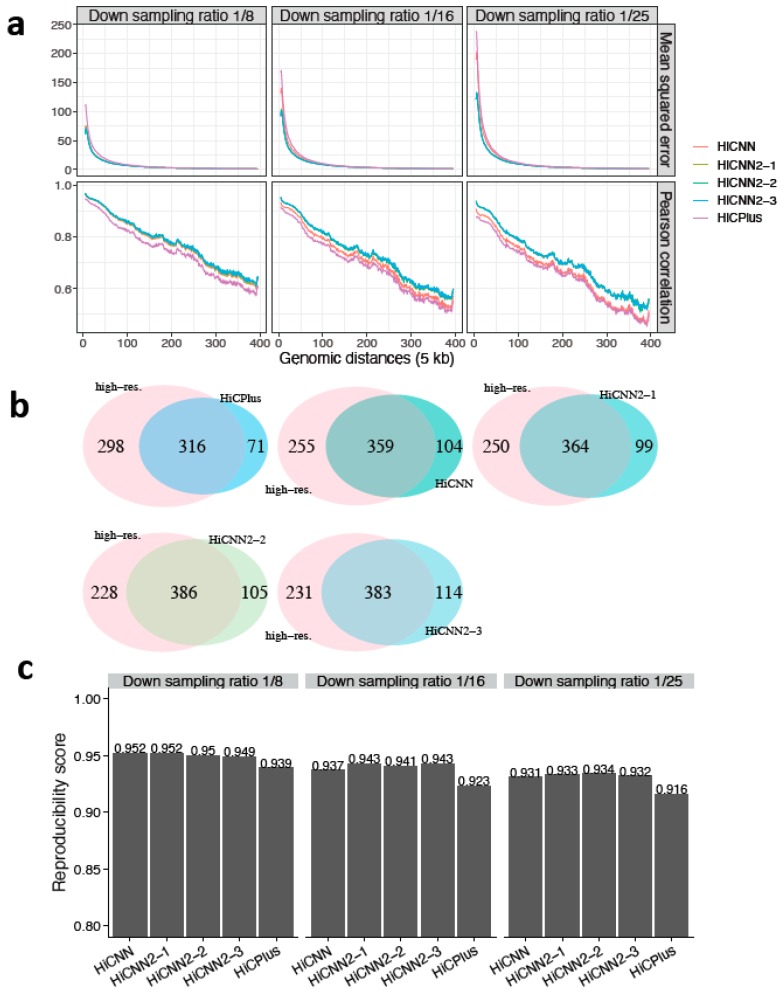
The evaluation results on chromosome 18 in mES cells between experimental high-resolution Hi-C (5 kb resolution) from Bonev lab and each of the five predicted Hi-C data sets, namely, HiCPlus-enhanced, HiCNN-enhanced, HiCNN2-1-enhanced, HiCNN2-2-enhanced, and HiCNN2-3-enhanced: (**a**) mean squared error and Pearson’s correlations with three different down-sampling ratios (1/8, 1/16, and 1/25); (**b**) the effectiveness of recovering significant interactions (detected by Fit-Hi-C with *q*-value < 0.05 within the genomic distances from 50 kb to 4 Mb) with a down-sampling ratio of 1/8; and (**c**) the reproducibility scores with the three down-sampling ratios.

We next explored whether our models, trained with Hi-C data in human GM12878, could be directly used in other species (e.g., mouse and bacteria). First, we generated three low-resolution Hi-C matrices on chromosome 18 of mouse embryonic stem cells (mES) by down-sampling the high-resolution (5 kb) Hi-C reads from Bonev lab with the three different down-sampling ratios. We then executed the five tools (HiCPlus, HiCNN, HiCNN2-1, HiCNN2-2, and HiCNN2-3) to improve the low-resolution matrices, with the evaluation results shown in Figure 4. The three HiCNN2 methods outperformed HiCNN and HiCPlus by achieving higher Pearson’s correlations and a smaller MSE. Fit-Hi-C was used to detect significant interactions (*q*-value < 0.05, genomic distances from 50 kb to 4 Mb). The three HiCNN2 methods (HiCNN2-2 for ratio 1/8, see Figure 4b; HiCNN2-1 for ratio 1/16, see Supplementary Figure S3a; and HiCNN2-3 for ratio 1/25, see Supplementary Figure S3b) achieved the maximum number of overlapping significant interactions with the real high-resolution Hi-C data. Furthermore, the three HiCNN2 methods achieved the highest reproducibility scores, as shown in Figure 4c, with the three different down-sampling ratios.

**Figure 5:**
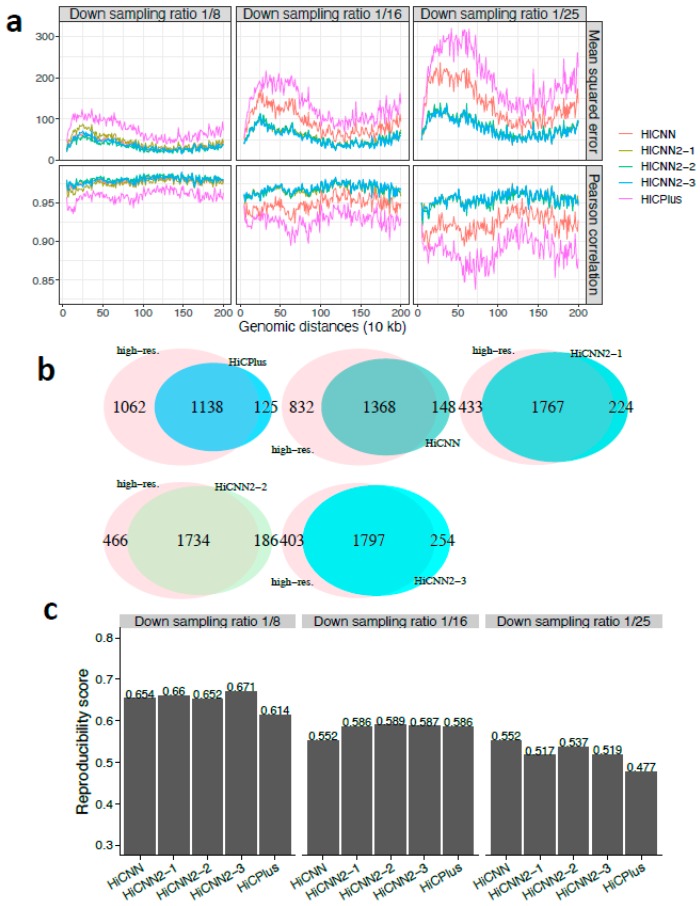
The evaluation results on the bacterial *C. crescentus* chromosome between experimental high-resolution Hi-C (10 kb resolution) and each of the five predicted Hi-C data sets, namely, HiCPlus-enhanced, HiCNN-enhanced, HiCNN2-1-enhanced, HiCNN2-2-enhanced, and HiCNN2-3-enhanced: (**a**) mean squared error and Pearson’s correlations with three different down-sampling ratios (1/8, 1/16, and 1/25); (**b**) the effectiveness of recovering significant interactions (detected by Fit-Hi-C with *q*-value < 0.05) with a down-sampling ratio of 1/25; and (**c**) the reproducibility scores with the three down-sampling ratios.

Furthermore, we generated three low-resolution Hi-C matrices of the bacterial *C. crescentus* chromosome by down-sampling its high-resolution (10 kb) matrix obtained from [25]. The MSE and Pearson’s correlation evaluation results are shown in Figure 5a, indicating that the three HiCNN2 methods outperformed HiCPlus and HiCNN. We can observe more obvious advantages of the three HiCNN2 methods with higher down-sampling ratios. In terms of recovering significant interactions detected by Fit-Hi-C (*q*-value < 0.05, genomic distances from 50 kb to 2 Mb), HiCNN2-2 for ratio 1/8 (Supplementary Figure S4a), HiCNN2-1 for ratio 1/16 (Supplementary Figure S4b), and HiCNN2-3 for ratio 1/25 (Figure 5b) performed the best among the five methods. The methods that obtained the highest reproducibility scores for the three different down-sampling ratios were HiCNN2-3, HiCNN2-2, and HiCNN, respectively, see Figure 5c. 

### 3.3. Enhancing Experimental Low-Resolution Hi-C Data on Human K562 and mES Cells

To test the effectiveness of enhancing real/experimental low-resolution Hi-C data, we used two experimental low-resolution Hi-C data sets to benchmark the methods. The first one was from Aiden lab [9] and was an independent Hi-C experiment (HIC071 from GEO GSM1551620) based on human K562 cells. The second one was from Ren lab [5] and based on mES cells.

**Figure 6:**
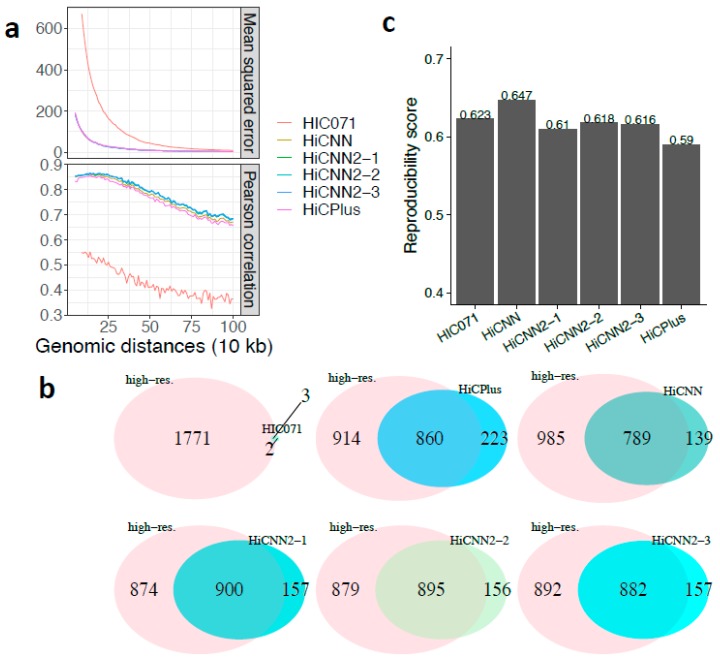
The evaluation results on chromosome 18 of human K562 cells between experimental high-resolution Hi-C (10 kb resolution) and each of the six Hi-C data sets, namely, real low-resolution (HIC071) from Aiden lab, HiCPlus-enhanced, HiCNN-enhanced, HiCNN2-1-enhanced, HiCNN2-2-enhanced, and HiCNN2-3-enhanced Hi-C data, with a down-sampling ratio of 1/16: (**a**) mean squared error and Pearson’s correlations; (**b**) the effectiveness of recovering significant interactions (detected by Fit-Hi-C with *q*-value < 0.05 within the genomic distances from 50 kb to 2 Mb); and (**c**) the reproducibility scores.

We enhanced the first low-resolution Hi-C data (HIC071) using the five methods on chromosome 18 with the down-sampling ratio equal to 1/16 and then compared the enhanced high-resolution matrices with the real high-resolution matrix. The MSE and Pearson’s correlation results shown in Figure 6a indicate that (1) the enhancement processes significantly improved the quality of HIC071 at 10 kb resolution and (2) the three HiCNN2 methods outperformed HiCPlus and HiCNN. The results in terms of the effectiveness of recovering significant interactions shown in Figure 6b indicate that (1) the enhancement processes successfully recovered more than 800 interactions compared with 3 in the original HIC071 Hi-C data set and (2) HiCNN2-1 performed the best, followed by HiCNN2-2 and HiCNN2-3. Notice that HiCNN achieved the highest reproducibility score, see Figure 6c.

In order to evaluate the methods on the second Hi-C data set, we considered the Hi-C data from Bonev lab [10] as real/experimental high-resolution (5 kb) Hi-C data in mES cells. The tests were conducted on chromosome 18 in mES cells with the down-sampling ratio equal to 1/8 with results shown in Supplementary Figure S5. Even though the two Hi-C data sets from Ren lab and Bonev lab were generated using different restriction enzymes, the five tools apparently improved the quality of the low-resolution data in terms of Pearson’s and Spearman correlations (Supplementary Figure S5a). The results in terms of significant interactions (*q*-value < 0.05, genomic distances from 50 kb to 4 Mb) are shown in Supplementary Figure S5b, indicating that HiCNN2-3 performed the best, followed by HiCNN2-2 and HiCNN2-1. The reproducibility scores shown in Supplementary Figure S5c indicate that the four methods (HiCNN, HiCNN2-1, HiCNN2-3, and HiCPlus) slightly improved the reproducibility by increasing the score from 0.32 to 0.36.

### 3.4. Recovering Topologically Associating Domains in Human IMR90 Cells

**Figure 7:**
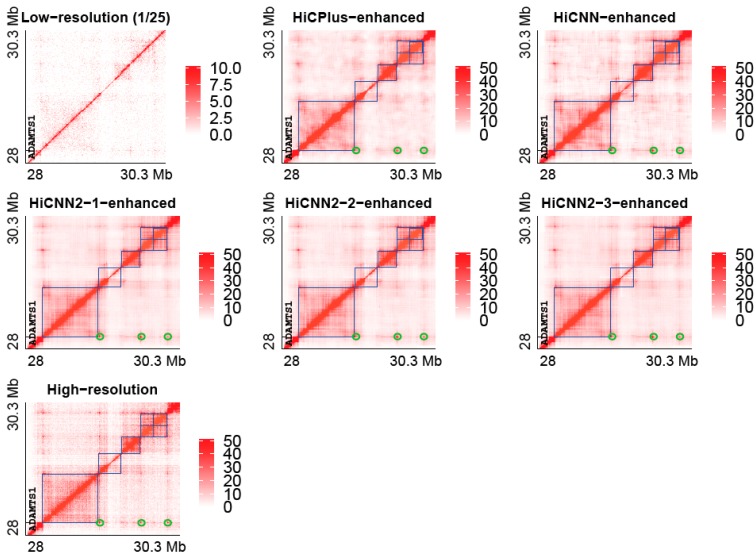
The heat maps of Hi-C contact matrices from low resolution (down sampling ratio 1/25), HiCPlus-enhanced, HiCNN-enhanced, HiCNN2-1-enhanced, HiCNN2-2-enhanced, and HiCNN2-3-enhanced, and real high-resolution Hi-C data on chromosome 21 (28–30.3 Mb) of human IMR90 cells. Six topologically associating domains (blue squares) and three Hi-C peaks (green circles) are highlighted in each of the predicted and real high-resolution heat maps (blue color). The models of the five methods were trained with input from GM12878 and with a down-sampling ratio equal to 1/25.

Topologically associating domains (TADs) defined in Hi-C contact matrices are important structural patterns of chromatins [5]. We explored whether computationally enhanced high-resolution data still preserve the boundaries of TADs. We plotted the heat maps of Hi-C contact matrices on chromosome 21 (28–30.3 Mb) of human IMR90 cells, see Figure 7. The following Hi-C contact matrices are presented in Figure 7: low resolution with a down-sampling ratio equal to 1/25, HiCPlus-enhanced, HiCNN-enhanced, HiCNN2-1-enhanced, HiCNN2-2-enhanced, HiCNN2-3-enhanced, and real high-resolution Hi-C data. We also highlighted the locations of six TADs detected by Arrowhead [9] on all of the predicted and real high-resolution heat maps (blue squares in Figure 7). It can be seen that the low-resolution Hi-C matrix was too sparse to be used to identify TAD locations. Compared to the low-resolution Hi-C matrix, the Hi-C matrices enhanced by the five computational tools (i.e., HiCPlus, HiCNN, HiCNN2-1, HiCNN2-2, and HiCNN2-3) are more similar to the real high-resolution Hi-C matrix, indicating that computational methods can help recover TAD patterns. Moreover, we can observe in Figure 7 that the tools can not only recover TAD boundaries but also reinforce Hi-C peaks (green circle) that are anchored at the promoter of the active *ADAMTS1* gene [9]. 

## 4. Conclusions

We developed HiCNN2, a computational package for improving the resolution of Hi-C data. HiCNN2 consists of three different architectures (i.e., HiCNN2-1, HiCNN2-2, and HiCNN2-3) using three different types of ConvNets. The first 56-layer ConvNet implements global and local residual learning and concatenates features from all local residual learning blocks. The second 22-layer ConvNet implements global residual learning. The last 3-layer ConvNet implements three traditional convolutional layers. Our evaluation results indicate that HiCNN2 consistently outperforms HiCNN and HiCPlus in terms of both predicting high-resolution Hi-C contacts and recovering significant genomic interactions. HiCNN2-1 is an updated version of our previously developed tool HiCNN; and our evaluations indicate that HiCNN2-1 significantly improves upon HiCNN. In general, the three architectures have their own advantages: HiCNN2-1 and HiCNN2-3 are recommended to be used when recovering more significant interactions is of interest, and HiCNN2-2 and HiCNN are the best choices if the goal is to achieve the highest reproducibility scores between the predicted and real high-resolution Hi-C matrices. HiCNN2 is freely available at http://dna.cs.miami.edu/HiCNN2/.

## Figures and Tables

**Table 1:** The performances of HiCNN2-1, HiCNN2-2, HiCNN2-3, HiCNN, and HiCPlus for recovering significant interactions detected by Fit-Hi-C (with three different *q*-values) that are in common with 306 Hi-C peaks detected by HiCCUPS on chromosome 17 in human GM12878. The best overlapping numbers are highlighted (bold numbers).

HiCCUPS	*q*-Value	High Resolution	HiCNN2-3	HICNN2-2	HICNN2-1	HiCNN	HiCPlus
306	<1 × 10^−6^	134	122	114	121	104	112
	<1 × 10^−3^	177	164	158	165	156	160
	<0.05	203	198	193	199	200	198

## Supplementary Materials

The following are available online at https://www.mdpi.com/2073-4425/10/11/862/s1, Figure S1: The effectiveness of recovering significant interactions (called by Fit-Hi-C with q-value < 0.05) on chromosome 17 in human GM12878 between experimental high-resolution Hi-C (10 kb) and each of the five predicted high-resolution Hi-C data sets, including HiCPlus-enhanced, HiCNN-enhanced, HiCNN2-1-enhanced, HiCNN2-2-enhanced, and HiCNN2-3-enhanced, with the down sampling ratios equal to 1/8 shown in (a) and 1/16 shown in (b), Figure S2: The effectiveness of recovering significant interactions (called by Fit-Hi-C with q-value < 0.05) on chromosome 10 in human K562 between experimental high-resolution Hi-C (10 kb) and each of the five predicted high-resolution Hi-C data sets, including HiCPlus-enhanced, HiCNN-enhanced, HiCNN2-1-enhanced, HiCNN2-2-enhanced, and HiCNN2-3-enhanced, with the down sampling ratios equal to 1/16 shown in (a) and 1/25 shown in (b), Figure S3: The effectiveness of recovering significant interactions (called by Fit-Hi-C with q-value < 0.05) on chromosome 18 in mES between experimental high-resolution Hi-C (5 kb) from Bonev lab and each of the five predicted high-resolution Hi-C data sets, including HiCPlus-enhanced, HiCNN-enhanced, HiCNN2-1-enhanced, HiCNN2-2-enhanced, and HiCNN2-3-enhanced, with the down sampling ratios equal to 1/16 shown in (a) and 1/25 shown in (b), Figure S4: The effectiveness of recovering significant interactions (called by Fit-Hi-C with q-value < 0.05) on a bacterial (C. crescentus) chromosome between experimental high-resolution Hi-C (10 kb) from Bonev lab and each of the five predicted high-resolution Hi-C data sets, including HiCPlus-enhanced, HiCNN-enhanced, HiCNN2-1-enhanced, HiCNN2-2-enhanced, and HiCNN2-3-enhanced, with the down sampling ratios equal to 1/8 shown in (a) and 1/16 shown in (b). The file name of the original high-resolution Hi-C matrix is “GSM1120445_Laublab_BglII_HiC_NA1000_swarmer_cell_untreated_replicate1_overlap_before_normalization.txt”, Figure S5: The evaluation results on chromosome 18 in mES between experimental high-resolution Hi-C (5 kb) from Bonev lab and each of the six Hi-C data sets, including real low-resolution from Ren lab, HiCPlus-enhanced, HiCNN-enhanced, HiCNN2-1-enhanced, HiCNN2-2-enhanced, and HiCNN2-3-enhanced Hi-C data with the down sampling ratio equal to 1/8: (a) the Pearson’s and Spearman correlations; (b) the effectiveness of recovering significant interactions (called by Fit-Hi-C with *q*-value < 0.05); and (c) the reproducibility scores.
