# Supplementary material for: HiCNN2: Enhancing the Resolution of Hi-C Data Using an Ensemble of Convolutional Neural Networks
Source: Genes (Basel). 2019 Oct 30;10(11):862. doi: 10.3390/genes10110862 (PMC6896157; doi:10.3390/genes10110862)
Supplement: Supplementary file 1 [file genes-10-00862-s001.pdf]

HiCNN2: Enhancing the Resolution of Hi-C Data Using an Ensemble of Convolutional Neural Networks

Supplementary Materials

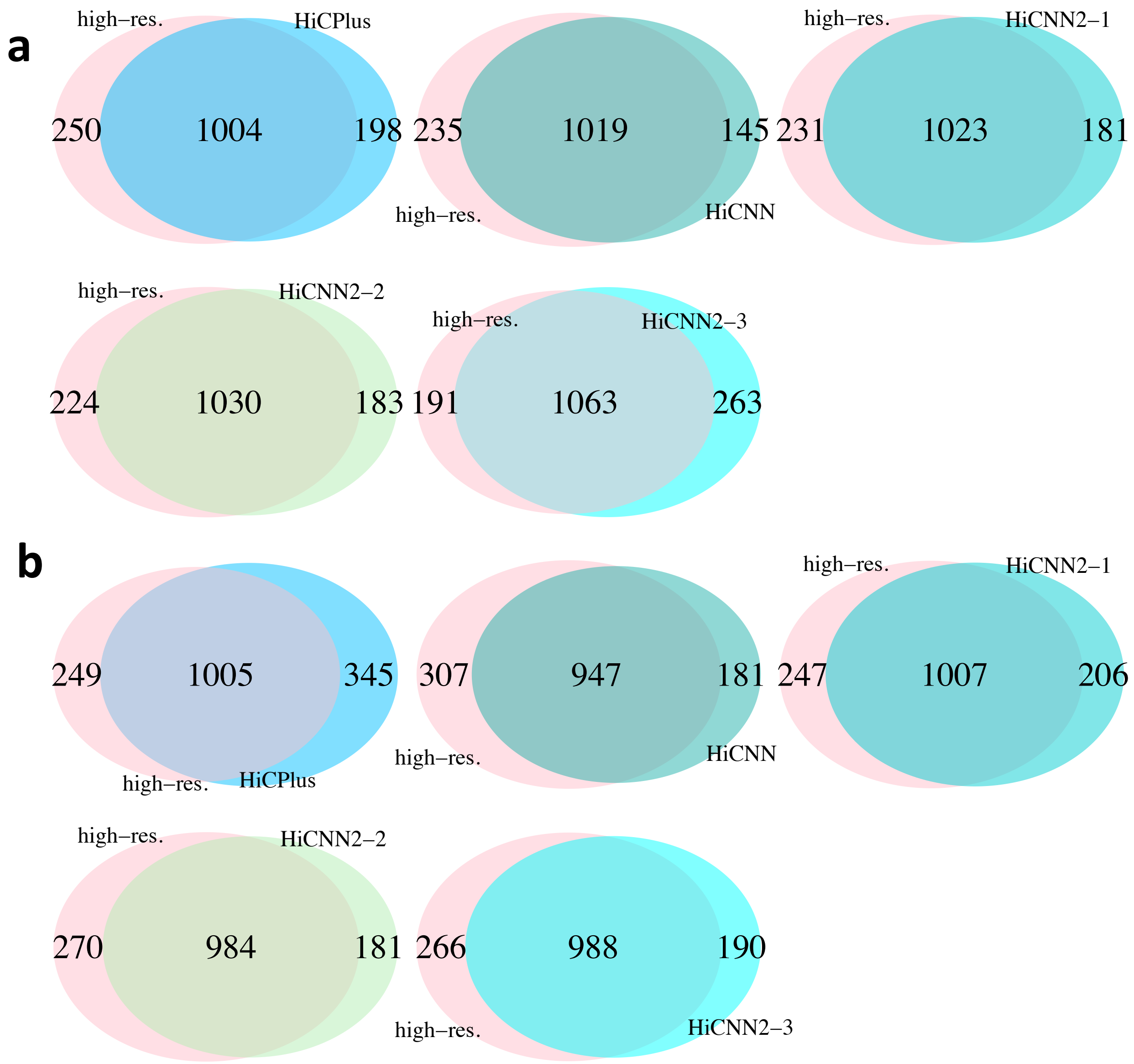

Figure S1. The effectiveness of recovering significant interactions (called by Fit-Hi-C with q-value < 0.05) on chromosome 17 in human GM12878 between experimental high-resolution Hi-C (10 kb) and each of the five predicted high-resolution Hi-C data sets, including HiCPlus-enhanced, HiCNN-enhanced, HiCNN2-1-enhanced, HiCNN2-2-enhanced, and HiCNN2-3-enhanced, with the down sampling ratios equal to 1/8 (a) and 1/16 (b).

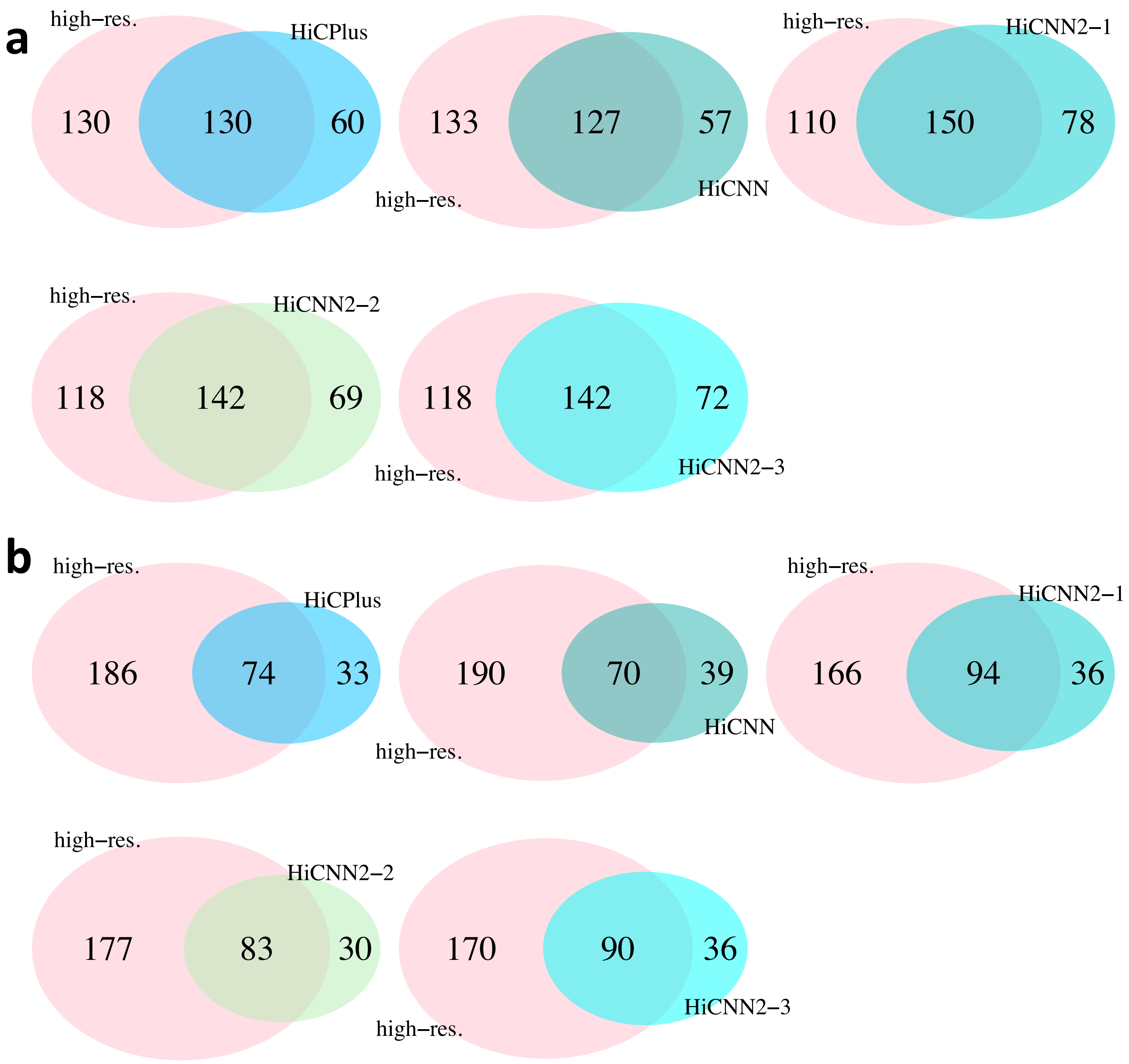

Figure S2. The effectiveness of recovering significant interactions (called by Fit-Hi-C with q-value < 0.05) on chromosome 10 in human K562 between experimental high-resolution Hi-C (10 kb) and each of the five predicted high-resolution Hi-C data sets, including HiCPlus-enhanced, HiCNN-enhanced, HiCNN2-1-enhanced, HiCNN2-2-enhanced, and HiCNN2-3-enhanced, with the down sampling ratios equal to 1/16 (a) and 1/25 (b).

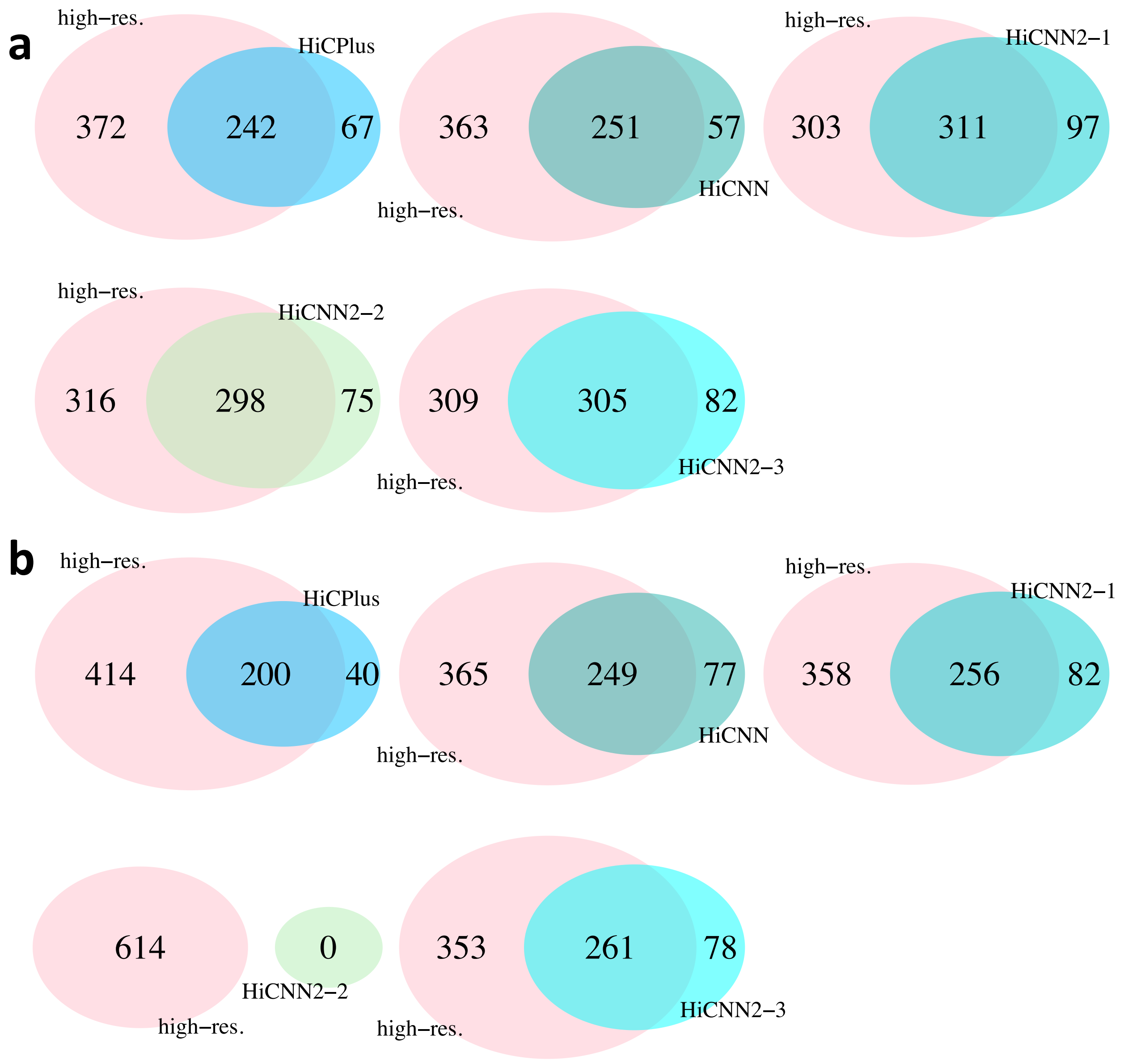

Figure S3. The effectiveness of recovering significant interactions (called by Fit-Hi-C with q-value < 0.05) on chromosome 18 in mES between experimental high-resolution Hi-C (5 kb) from Bonev lab and each of the five predicted high-resolution Hi-C data sets, including HiCPlus-enhanced, HiCNN-enhanced, HiCNN2-1-enhanced, HiCNN2-2-enhanced, and HiCNN2-3-enhanced, with the down sampling ratios equal to 1/16 (a) and 1/25 (b).

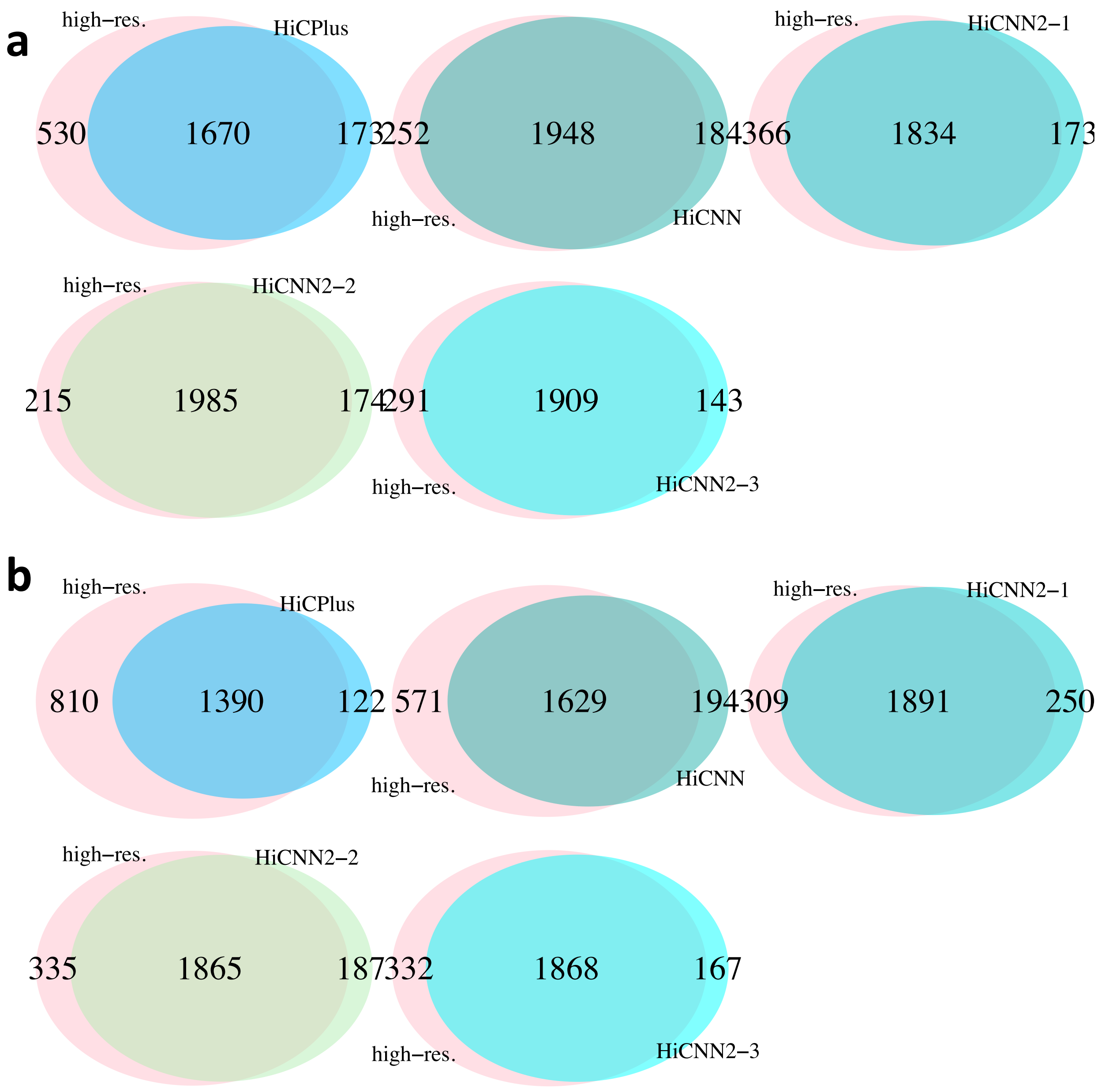

Figure S4. The effectiveness of recovering significant interactions (called by Fit-Hi-C with q-value < 0.05) on a bacterial (Caulobacter crescentus) chromosome between experimental high-resolution Hi-C (10 kb) from Bonev lab and each of the five predicted high-resolution Hi-C data sets, including HiCPlus-enhanced, HiCNN-enhanced, HiCNN2-1-enhanced, HiCNN2-2-enhanced, and HiCNN2-3-enhanced, with the down sampling ratios equal to 1/8 (a) and 1/16 (b). The file name of the original high-resolution Hi-C matrix is "GSM1120445_Laublab_BglII_HiC_NA1000_swarmer_cell_untreated_replicate1_overlap_before_normalization.txt".

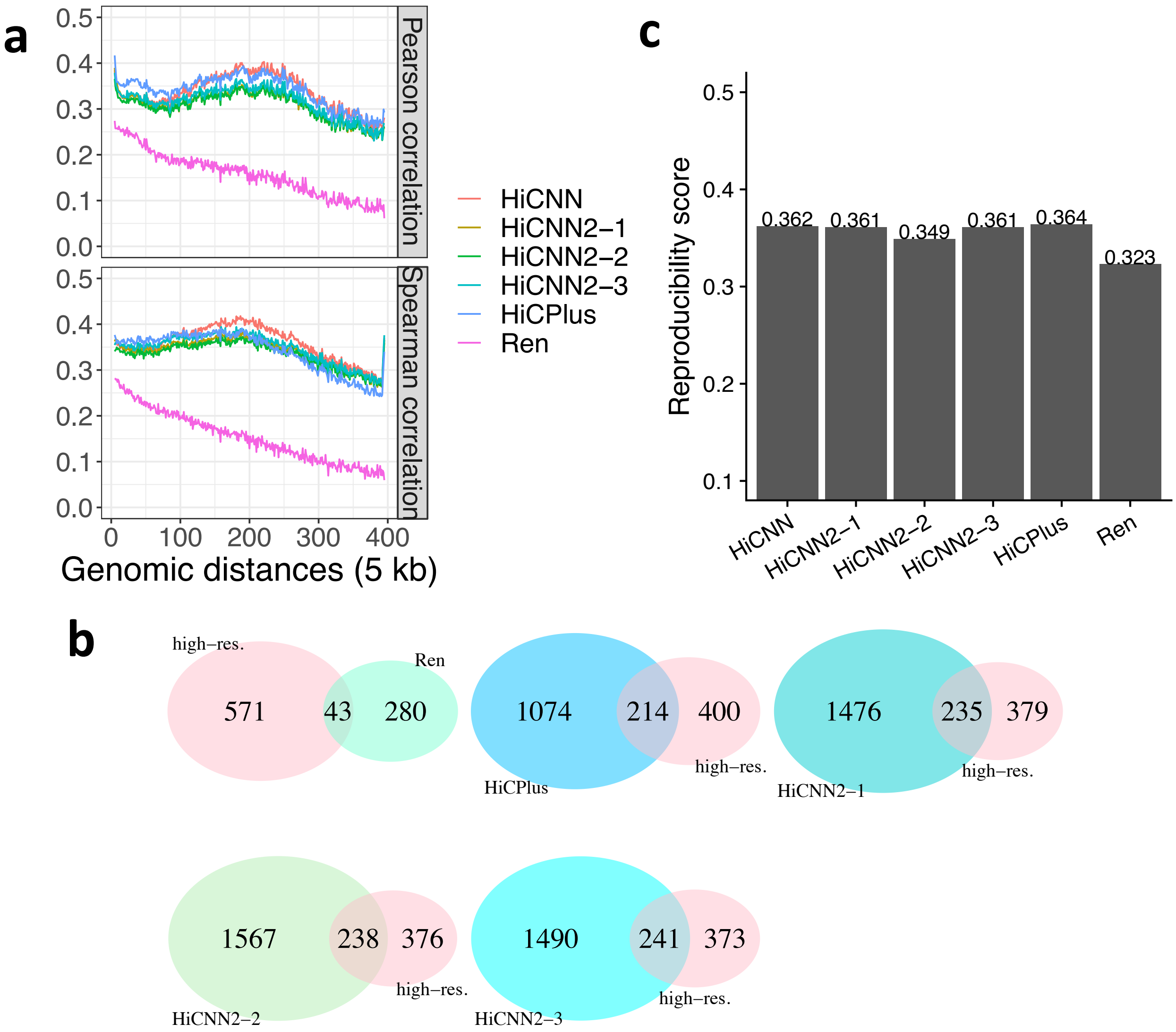

Figure S5. The evaluation results on chromosome 18 in mES between experimental high-resolution Hi-C (5 kb) from Bonev lab and each of the six Hi-C data sets, including real low-resolution from Ren lab, HiCPlus-enhanced, HiCNN-enhanced, HiCNN2-1-enhanced, HiCNN2-2-enhanced, and HiCNN2-3-enhanced Hi-C data with the down sampling ratio equal to 1/8: (a) the Pearson's and Spearman correlations; (b) the effectiveness of recovering significant interactions (called by Fit-Hi-C with q-value < 0.05); and (c) the reproducibility scores.
